# Genotyping Strategies Using ddRAD Sequencing in Farmed Arctic Charr (*Salvelinus alpinus*)

**DOI:** 10.3390/ani11030899

**Published:** 2021-03-21

**Authors:** Fotis Pappas, Christos Palaiokostas

**Affiliations:** Department of Animal Breeding and Genetics, Swedish University of Agricultural Sciences, P.O. Box 7090, 750 07 Uppsala, Sweden; foas0001@stud.slu.se

**Keywords:** reduced representation sequencing, *Salvelinus alpinus*, selective breeding, GBS, salmonid

## Abstract

**Simple Summary:**

Animal breeding in recent years has benefited greatly from the availability of large-scale genetic information. The most widely applied genomic tools in selective breeding are specialized arrays that use DNA hybridization. However, the high financial investments accompanying this practice impair the profitability of emerging aquaculture species, including Arctic charr. The aim of the current study was to assess and compare the potential of two cost-efficient genotyping strategies applicable in a variety of breeding-related tasks, such as pedigree verification, genetic diversity screening and detection of genomic regions that are associated with phenotypes of economic importance. Both strategies are based on reduced representation sequencing but differ in sequencing coverage (low and high). The low coverage strategy offers a higher density of DNA markers, but also presents a greater proportion of missing data in the marker set and is characterized by more uncertainty in determining heterozygosity compared to high coverage. Our results show that while high coverage genotyping performs better in genetic diversity and kinship analyses, a low coverage strategy is more successful in identifying genomic regions associated with phenotypic traits, leading to the conclusion that both strategies could be of value into selection schemes.

**Abstract:**

Incorporation of genomic technologies into fish breeding programs is a modern reality, promising substantial advances regarding the accuracy of selection, monitoring the genetic diversity and pedigree record verification. Single nucleotide polymorphism (SNP) arrays are the most commonly used genomic tool, but the investments required make them unsustainable for emerging species, such as Arctic charr (*Salvelinus alpinus*), where production volume is low. The requirement to genotype a large number of animals for breeding practices necessitates cost effective genotyping approaches. In the current study, we used double digest restriction site-associated DNA (ddRAD) sequencing of either high or low coverage to genotype Arctic charr from the Swedish national breeding program and performed analytical procedures to assess their utility in a range of tasks. SNPs were identified and used for deciphering the genetic structure of the studied population, estimating genomic relationships and implementing an association study for growth-related traits. Missing information and underestimation of heterozygosity in the low coverage set were limiting factors in genetic diversity and genomic relationship analyses, where high coverage performed notably better. On the other hand, the high coverage dataset proved to be valuable when it comes to identifying loci that are associated with phenotypic traits of interest. In general, both genotyping strategies offer sustainable alternatives to hybridization-based genotyping platforms and show potential for applications in aquaculture selective breeding.

## 1. Introduction

Over the last decade, genomic technologies have transformed the field of aquaculture, opening up exciting new avenues for in-depth studies of practically any trait of interest [1]. Implementations of genomic technologies in aquaculture breeding schemes relying on single nucleotide polymorphism (SNP) arrays have been most beneficial for the major aquaculture species [2]. Nevertheless, SNP arrays require considerable financial investments and running costs that are particularly difficult to sustain in the case of rare aquaculture species. Reduced-representation genotyping platforms offer a cost-effective solution for detecting genome-wide genetic polymorphisms irrespective of the availability of prior genomic information [3]. An abundance of reduced-representation based methodologies have become available over the last years following the introduction of restriction site-associated DNA (RAD) sequencing [4].

The RAD-derived platforms supported by a plethora of available restriction enzymes have proven to be particularly flexible in their applications [5]. Most commonly used type II restriction enzymes recognize between 4 and 8 bases of sequence motifs. Since the sequencing output per lane and the associated cost are in general fixed for each platform (minimal variation between sequencing runs), a balance needs to be obtained between genotyping density and sequencing cost per sample. Naturally, more frequent enzymatic cutters will result in higher genotyping densities. On the other hand, the required sequencing effort, and as such, the associated cost per sample for identifying genomic polymorphisms increase in correspondence with the number of interrogated sites [6]. In comparison, the choice of less frequent enzymatic cutters would allow multiplexing a higher number of animals in a sequencing lane, reducing the genotyping cost per sample at the expense though of reducing the obtained genotyping density.

A number of common enzymatic cutters used for genotyping by sequencing (GBS) [7] have been successfully applied in the form of low read coverage per interrogated site for deciphering genomic relationships at a high resolution [8]. Low coverage genotyping by sequencing has been successfully applied for estimating genome-wide linkage disequilibrium [9], genetic map construction [10], gender prediction [11], parentage assignment [12] and genomic selection purposes [13,14]. Nevertheless, the aforementioned strategy usually results in an increase of missing genotypic data and in a reduction of confidently identifying heterozygotes due to the low number of supporting reads as opposed to high coverage genotyping approaches.

Arctic charr (*Salvelinus alpinus*) is an attractive candidate for diversifying the Nordic aquaculture industry, with an ongoing breeding program having been operational in Sweden for approximately 40 years [15,16]. Nevertheless, limited implementation of modern genomic technologies has taken place to guide selection decisions. Double digest RAD (ddRAD) relying on the simultaneous usage of two enzymes is one of the most popular reduced-representation sequencing platforms. The combination of a relatively easy library construction workflow and cost-efficiency [6] makes ddRAD particularly useful for studying the genetic diversity of populations [17,18,19,20], constructing genetic maps [21,22,23,24] and quantitative trait loci mapping [25,26,27,28,29].

In the current study, we compared two genotyping strategies based on ddRAD that were applied on 277 (ten full-sibling families) and 188 (eight full-sibling families; subset of the 277 samples) samples, respectively, of Arctic charr originating from the national Swedish breeding program. During the first genotyping scenario, we applied less frequent cutting restriction enzymes (SbfI-SphI) while aiming to identify SNPs supported by high read coverage. In the second scenario, we constructed shallow coverage ddRAD libraries through the usage of more frequent enzymatic cutters (PstI-NlaIII). SNPs were identified under both scenarios and were used for estimating genetic diversity metrics, genomic relationships, and identifying growth-related quantitative trait loci (QTLs).

## 2. Materials and Methods

### 2.1. Sample Background and Phenotypes

Arctic charr from the national Swedish breeding program was used for the needs of our study. The animals were located at the facilities of Aquaculture Center North (ACN) in Kälarne, Sweden. Growth measurements (body weight and total length) were recorded from 277 animals (10 months old) of the 2017 class, representing 10 full-sibling families (with two families having the same sire). In addition, the condition factor (K) was calculated for all the genotyped animals using the formula K = 10^5^ × weight/length^3^. Finally, fin-clips were collected for DNA extraction and ddRAD library preparation.

### 2.2. DNA Extraction and Quantification

Fin clips of Arctic charr of about 3 mm^2^ were collected and preserved in 100% ethanol. Genomic DNA from individual samples was extracted using a salt precipitation method [30] and eluted in 30 μL of 5 mM Tris. The quality of the obtained DNA was assessed through gel electrophoresis (1% agarose gel). DNA samples were quantified using a Qubit fluorimeter (Thermo Fisher Scientific, Waltham, MA, USA) and diluted with Tris buffer (5 mM) to 15 ng/μL.

### 2.3. ddRAD Library Preparation and Sequencing

ddRAD libraries were simulated in silico using RADinitio v1.1.1 [31] and were prepared according to a standard protocol [6], with minor modifications that have been previously described [32]. Two different types of ddRAD libraries were prepared using either less frequent (SbfI-SphI; *n* = 277; File S1) or more frequent enzymatic cutters (PstI-NlaIII; *n* = 188; File S2). Briefly, each sample (15 ng/μL DNA) was digested at 37 °C for 60 min in the same reaction with either SbfI (recognizing the CCTGCA|GG motif) and SphI (recognizing the GCATG|C motif) or PstI (recognizing the CTGCA|G motif) and NlaIII (recognizing the CATG|N motif) high fidelity restriction enzymes (New England Biolabs, UK; NEB), by using 6 U of each enzyme per microgram of genomic DNA in 1× Reaction Buffer 4 (NEB). The reactions (6 μL final volume) were then heat inactivated at 65 °C for 20 min. Individual-specific combinations of P1 and P2 adapters, each with a unique 5 or 7 base pair (bp) barcode, were ligated to the digested DNA at 22 °C for 120 min by adding 1 μL SbfI-PstI compatible P1 adapter (25 nM), 0.7 μL SphI-NlaIII compatible P2 adapter (100 nM), 0.06 μL 100 mmol/L rATP (Promega, Southampton, UK), 0.95 μL 1× Reaction Buffer 2 (NEB), 0.05 μL T4 ligase (NEB, 2 × 106 U/mL) and reaction volumes made up to 12 μL with nuclease-free water for each sample. Following heat inactivation at 65 °C for 20 min, the ligation reactions were slowly cooled to room temperature (over 1 h) then combined in a single pool (for one sequencing lane) and purified. Size selection (300–600 bp) was performed by agarose gel separation and followed by gel purification and PCR amplification. A total of 100 μL each of the amplified libraries (12–14 PCR cycles) was purified using an equal volume of AMPure beads. The libraries were eluted into 20 μL EB buffer (MinElute Gel Purification Kit, Qiagen, Chadstone, Australia). The libraries were sequenced on two SP flow cells of an Illumina NovaSeq6000 using 300 cycles and v1.5 chemistry kits (150 bp paired end-reads) at the National Genomics Infrastructure in Uppsala, Sweden.

### 2.4. Sequence Data Analysis and SNP Genotyping

Reads of low quality (Q < 20) and missing the expected restriction sites were discarded. The retained reads were aligned to the *Salvelinus* sp. reference genome assembly GCA_002910315.2 using bowtie2 [33]. Stacks v2.5 [34] was used to identify and extract single nucleotide polymorphisms (SNPs) using the *gstacks* module. An alpha threshold of 0.01 and 0.05 for calling SNPs and genotypes respectively was applied using the *marukilow* model of *gstacks*. From each putative ddRAD locus, only a single SNP was used for downstream analysis. SNPs with minor allele frequency (MAF) < 0.05 and maximum heterozygosity > 0.7 across the tested samples were discarded using the *populations* module of Stacks. Only SNPs found in at least 50% and 70% of the samples in the low and high coverage datasets respectively were retained for downstream analysis.

### 2.5. Genetic Diversity and Kinship

For the direct comparison of the two genotyping strategies, the intersecting population (individuals present in both SbfI-SphI and PstI-NlaIII filtered datasets; *n* = 175) was used. Expected (He) and observed (Ho) heterozygosity metrics were calculated separately for the two datasets using the R package adegenet 2.1.3 [35]. Principal component analysis (PCA) was performed for both genotyping scenarios with the aforementioned software to gain information regarding the population structure. Additionally, discriminant analysis of principal components (DAPC) was conducted to further investigate the presence of genetic clusters. The optimal number of clusters (K) was selected for each dataset using the Bayesian Information Criterion (BIC) [36]. Furthermore, a cross-validation scheme was performed to test the ability of the two marker sets to discriminate between individuals from different full-sibling families. More specifically, data from ~75% of the individuals were used as training sets and the rest were utilized as testing sets (one family was excluded since after quality control it consisted of only 2 members). Information derived from DAPC (*predict.dapc*) performed on the training data sets was used to predict the family of origin for the members of the testing sets and assignment accuracies were calculated. Finally, in order to determine the effect of MAF on the reassignment performance of the low coverage set, we applied a series of MAF filters (0.05, 0.1, 0.15, 0.2 and 0.25) and subjected the yielded sets to the same cross-validation method.

The intersecting population was also used to compute genomic relationship matrices (GRMs) and compare the two datasets in regard to kinship estimations among full siblings. For this purpose, we employed R/rrblup 4.6.1 [37,38] and calculated the GRMs for each dataset setting a threshold of 0.3 for maximum missing data and using the Expectation-Maximization (EM) algorithm [39]. Genomic relatedness for full-sibling and non-sibling pairs was then expressed using the following formula:

(1) $${\mathrm{GR}}_{\mathrm{ij}}=\frac{g_{\mathrm{ij}}}{\sqrt{g_{\mathrm{ii}}\cdot g_{\mathrm{jj}}}}$$

where GR_ij_ is the genomic relatedness between individuals i and j, and g refers to the genomic relationships as calculated in the GRMs mentioned above.

### 2.6. Association with Phenotypic Traits

Association studies for the recorded growth traits were performed separately for the two genotyping strategies and their respective populations. Analyses were performed using the R package gaston 1.5.5 [40]. The tests were carried out aiming to identify genomic regions associated with body length and log_2_ transformed K factor. A linear mixed model was applied:

**Y = Xα + Ζβ + Sω + ε** (2)

where **α** is the vector of fixed effects other than marker effects (intercept, sex, family-tank), **β** is the fixed effect of each SNP marker, **ω** is the vector of animal random effect ~N (0, **Gσ_α_^2^**) and **ε** is the vector of residuals ~N (0, **Iσ_e_^2^**). **X**, **Z** and **S** are incidence matrices relating **Y** with **α** and **β** respectively. **G** is a genomic relationship matrix computed with the *GRM* function, **I** is an identity matrix, **σ_e_^2^** is the residual variance and **σ_α_^2^** represents the additive genetic variance. Multi-test adjustment of the genome-wide significance thresholds was achieved using Bonferroni corrections (0.05α level/*n*), where n is the number of tested SNPs. Furthermore, Benjamini–Hochberg (BH) adjustment of *p*-values was performed to control for false discovery rate.

## 3. Results

### 3.1. Genotypic Information

**Figure 1:**
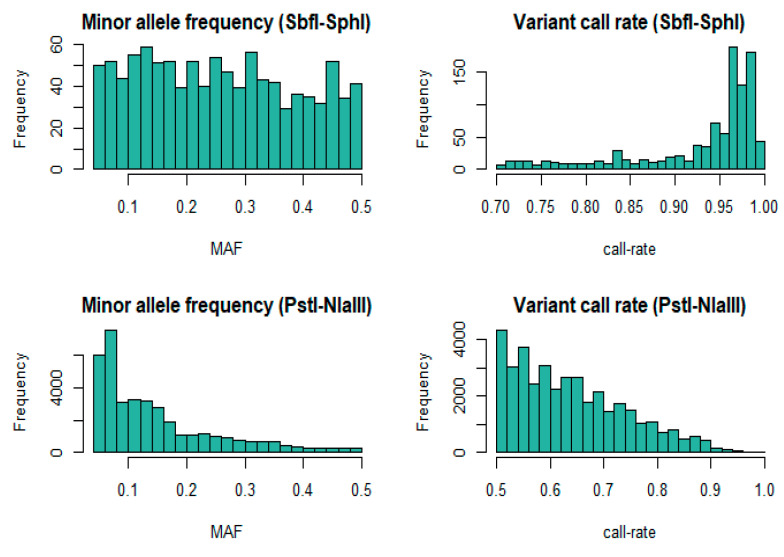
Distributions of post-filtering minor allele frequency (MAF) and single nucleotide polymorphism (SNP) call rate for SbfI-SphI (*n* = 253) and the intersecting (*n* = 175) animals that were also genotyped with PstI-NlaIII.

Due to high rate of missing data, 24 animals from the high coverage dataset (HC) and nine animals from the low coverage dataset (LC) were removed. Therefore, the HC dataset was comprised of 253 animals, and 179 were finally represented in the LC dataset. The number of individuals successfully genotyped with both SbfI-SphI and PstI-NlaII was 175 (intersecting population) and was used for analyses where direct comparisons were necessary. The expected number or RAD loci as estimated with the in-silico library stimulations was 1524 for SbfI-SphI (HC) and 305,077 for PstI-NlaIII (LC). In contrast, the filtered variant sets in our study consisted of 1034 SNP genotypes for SbfI-SphI, and 38,224 SNPs for PstI-NlaIII. The mean coverage for the former was 428X, while for the latter the mean coverage was 3X. The distributions of MAF and SNP call rates after filtering are shown in Figure 1. The marker set derived from the shallow coverage approach was characterized by a greater proportion of low call-rate genotypes and MAF compared to high coverage.

### 3.2. Descriptive Statistics of Phenotypic Traits

**Figure 2:**
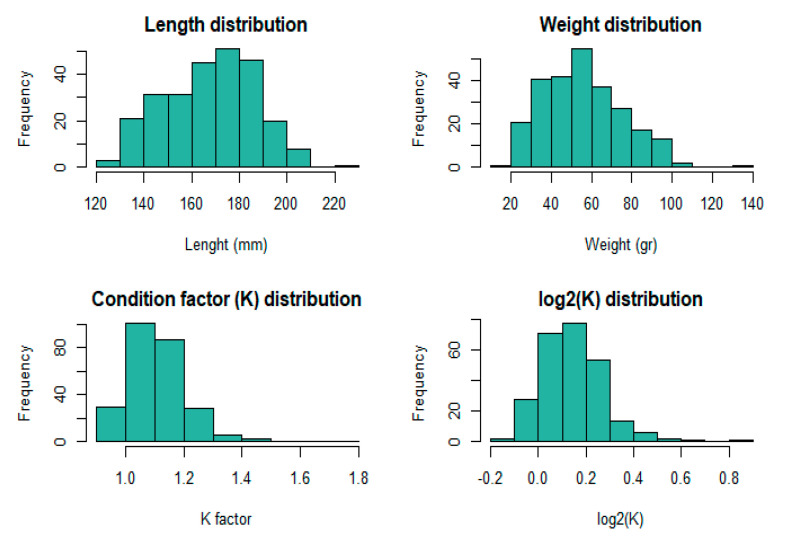
Histograms of distributions for length, weight, K factor and log_2_ transformed K factor (*n* = 257).

The distributions of phenotypic data are graphically presented in Figure 2. The overall mean body length of the studied sample was 169.03 mm (sd = 19.01), and the mean wet body weight was 55.88 gr (sd = 19.57). For condition factor K, the mean value was 1.11 (sd = 0.11) and a log_2_ transformation was performed to obtain a normalized distribution (Figure 2).

### 3.3. Genetic Diversity

**Figure 3:**
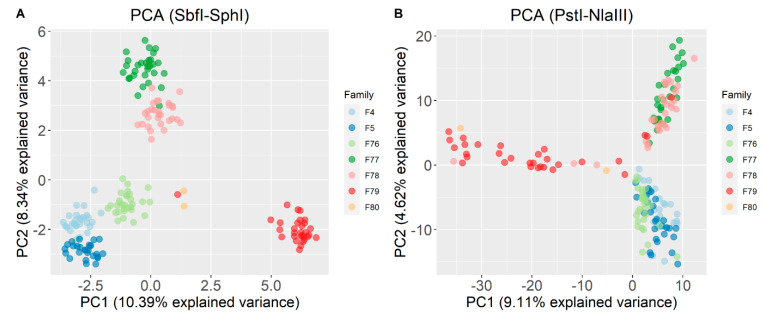
Principal component analysis for (**A**) SbfI-SphI and (**B**) PstI-NlaIII genotyping scenarios. The represented population is the intersection (*n* = 175) of the individuals that were genotyped in both scenarios.

For the high coverage dataset, the expected (He) and observed (Ho) heterozygosity were 0.34 and 0.31 respectively; and the same metrics for the shallow coverage scenario were 0.23 and 0.22. Differences in subpopulations detection were visualized when dimensionality reduction was used through PCA (Figure 3). The first and second principal components for the SbfI-SphI SNP set accounted for 10.39% and 8.34% of the observed variation, while for the PstI-NlaIII dataset, the proportions for PC1 and PC2 were 9.11% and 4.62% respectively.

**Figure 4:**
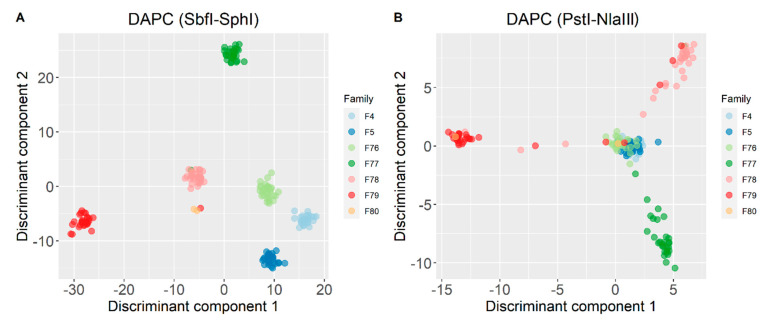
Discriminant analysis of principal components (DAPC) for (**A**) SbfI-SphI and (**B**) PstI-NlaIII genotyping scenarios. The represented population is the intersection (*n* = 175) of the individuals that were genotyped in both scenarios.

DAPC provided further insights of the formed genetic clusters (Figure 4). The optimal number of clusters (K) was suggested to be seven for SbfI-SphI and five for PstI-NlaIII. In general, clustering according to the shallow coverage dataset was less efficient and only provided a rough discrimination between the different families. In the analyses of the high coverage SNP sets, two individuals (belonging to families F77 and F79) failed to group with their original families and were assigned to closely related groups (Figure 4A). Finally, the genetic clusters representing families F4 and F5, which had the same sire, always appeared to group closely (SbfI-SphI) or were indistinguishable (PstI-NlaIII).

**Figure 5:**
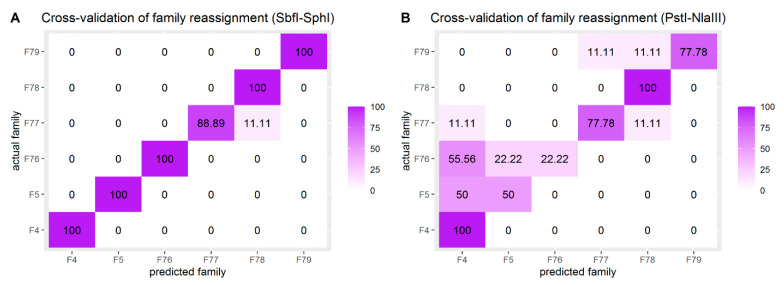
Heatmaps visualizing the relative frequencies (%) of family predictions according to the DAPC cross-validation scheme for (**A**) SbfI-SphI and (**B**) PstI-NlaIII.

A cross-validation scheme was employed to assess the accuracy of family reassignment (DAPC) using the two datasets. The relative frequency (observed minimum) of assignment to the original (according to pedigree records) family was 95.83% for SbfI-SphI and 68.75% for PstI-NlaIII. Frequencies of family assignments as calculated during cross-validation are visualized in Figure 5 for both SNP sets. Furthermore, the analysis performed on PstI-NlaIII dataset demonstrated that reassignment accuracy remained at the same levels for MAF filtering thresholds spanning from 0.05 to 0.25 (Figure S1).

**Figure 6:**
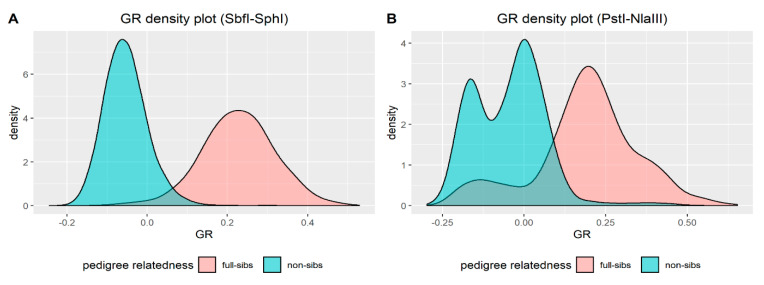
Density plots of genomic relatedness (GR) among full siblings and non-siblings for: (**A**) SbfI-SphI and (**B**) PstI-NlaIII.

Mean genomic relatedness (see Genetic Diversity and Kinship in Materials and Methods) among full siblings was found to be 0.23 (sd = 0.09) for SbfI-SphI and 0.19 (sd = 0.16) for PstI-NlaIII, and the same metric for non-sibling pairs was −0.05 (sd = 0.06) for SbfI-SphI and −0.05 (sd = 0.11) for PstI-NlaIII. The density plots in Figure 6 visualize the overlapping of GRs distributions between full-sibling and non-sibling pairs, which was found to be 3.70% for SbfI-SphI and 16.08% for PstI-NlaIII.

### 3.4. Association Scans for Phenotypic Traits

**Figure 7:**
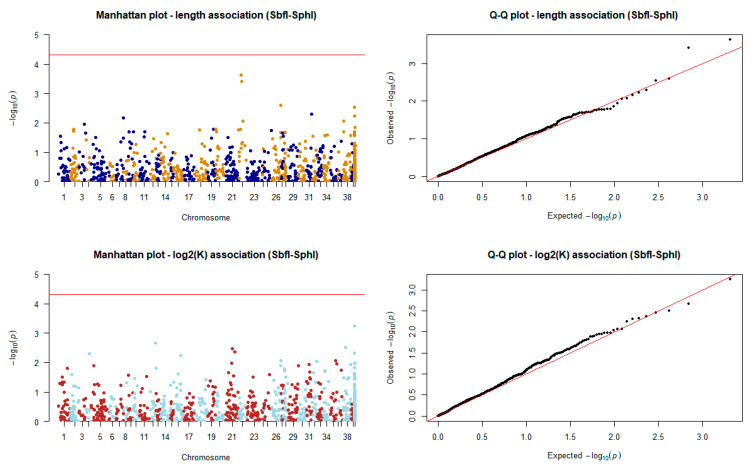
Manhattan and quantile–quantile plots of the association tests for length and log_2_K in the SbfI-SphI scenario (*n* = 253). The red horizontal line indicates the Bonferroni error rate-adjusted significance level.

No significant QTL peaks were observed for body length or log_2_ transformed condition factor (K) when using genotypic information from the high coverage dataset (Figure 7).

**Figure 8:**
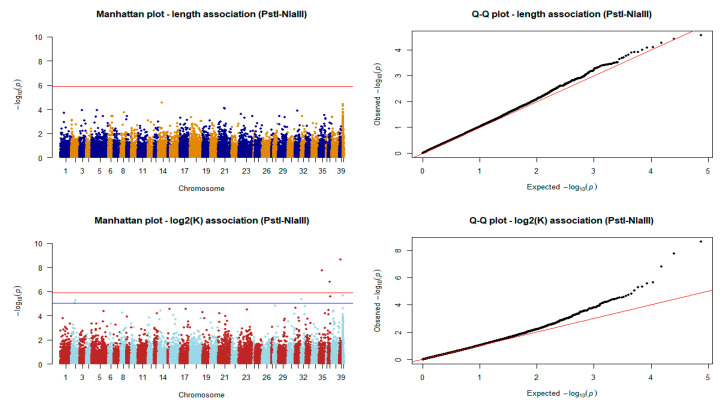
Manhattan and quantile–quantile plots of the association tests for length and log_2_K in the PstI-NlaIII scenario (*n* = 179). The red horizontal line indicates the Bonferroni error rate-adjusted significance level. The blue line indicates the threshold of the significant markers after BH adjustment of *p*-values.

Association of markers derived from the shallow coverage strategy with body length failed to identify QTLs. Nevertheless, SNPs from the same dataset were found to be significant in the case of log_2_K. As shown in Figure 8, a total of eight SNPs were identified. More specifically, three SNP markers passed the Bonferroni adjusted significance threshold, and another five SNPs were found to be significantly associated with the log_2_ transformed condition factor after adjusting the obtained *p*-values with the BH method.

Finally, we highlight 11 genes based on physical distance (less than 20 kb) from the chromosomal position of each variant (Table 1) and found that three of these SNPs were located in genes, while two others were located within a distance of less than 1000 bp from genes.

## 4. Discussion

In the current study, both high and low coverage ddRAD genotyping scenarios provided genotypic information that is useful for different applications in a wide range of breeding related tasks. Reduced representation sequencing can be a valuable tool for selective breeding practices in emerging aquaculture species, such as Arctic charr [14]. Genomic information is useful for important aspects of fish breeding that include, but are not limited to estimating relationships between individuals, assessing genetic diversity in populations and even investigating the genetic basis of phenotypic variation [41]. However, the cost of genotyping is a limiting factor for the sustainability of breeding programs. Available, reduced representation methods include strategies that yield for the same cost either deep coverage but low density of genetic markers, or high genotyping density with shallow coverage—and thus, a greater proportion of missing data. Encouragingly, as suggested by both simulated data and empirical studies, low coverage genotyping by sequencing appears to be an attractive option for selective breeding practices [12,42].

As expected by RADinitio library simulations, the PstI-NlaIII library resulted in significantly higher number of SNP biallelic genotypes than SbfI-SphI. The actual counts of filtered variants were lower than estimated in silico with the deviation being larger and more profound for the low coverage scenario. This could be attributed to a number of factors that among others include the low depth in the case of PstI-NlaIII (~ 3X) and the fact that the reference genome was derived from a distant population and might not be adequately representative of the nucleus of the national Swedish breeding program.

### 4.1. Genetic Diversity

The deep coverage strategy outperformed shallow coverage genotyping. A comparison of heterozygosity metrics indicates that the latter underestimates the proportion of heterozygotes in the population, possibly due to missing information in the marker set [10]. Observed heterozygosity (Ho) was found to be lower than expected heterozygosity (He) in both cases, reflecting the fact that the studied population was derived from a closed-nucleus breeding program [43].

Furthermore, genotyping in the SbfI-SphI scenario resulted in better and more robust detection of subpopulations and clustering at the family level in the PCA and DAPC analytical procedures. This finding was further supported by a cross-validation scheme where the estimated accuracy of family reassignment was higher for deep coverage, suggesting that high quality genotypic information is a priority over marker density for genetic diversity and population structure analysis. Despite providing a much more defined view on genetic clusters, DAPC for the SbfI-SphI scenario resulted in two individuals not grouping with their original families, but this could be explained by putative errors in the pedigree records.

It should be highlighted that in our study we tried to decipher the population structures and genetic clustering of full-sibling families in a closed breeding nucleus, a task that is more challenging and demanding than studying genetic diversity and differentiation among distant populations [44]. Even though differing MAF thresholds could potentially affect population structure inference [45,46], setting stricter MAF filtering thresholds for the PstI-NlaIII dataset in our study did not affect the clustering performance. However, the total number of SNP markers decreased, which could consequently result in decreased computational intensity.

### 4.2. Kinship Investigation

Kinship estimations among full-sibling pairs were higher for the high coverage dataset and provided better discrimination from the GR values of non-sibling pairs, despite incorporating remarkably less genotypic information than the low coverage dataset. As with genetic diversity analyses, this might have been due to the higher SNP calling uncertainty entailed by shallow coverage genotyping-by-sequencing approaches. Overall, both genotypic datasets underestimated the average relationship between full-sibling pairs, thereby performing worse compared to similar studies in livestock that used SNP arrays [47]. The above was probably to be expected in the case of HC dataset, since the number of SNPs (~1000) is considered low for providing the resolution needed for a genomic relationship matrix. Notably, genomic relationship matrices are most commonly constructed from SNPs in the range of tens of thousands [48]. On the other hand, a high-density SNP dataset even at low coverage is expected to result in a more accurate genomic relationship matrix and better discern relationships between full siblings. More specifically, low coverage genotyping by sequencing has been shown to be effective in estimating genomic relationships amongst full siblings both in Atlantic salmon [8] and in Arctic charr [14]. However, compared to the above studies the library preparation protocol of our study differed. In particular, opposed to the previous studies, the size selection during library preparation was performed manually which might have rendered the detection of SNPs in the low coverage dataset more challenging due to the larger number of sampled sites. As a result, the LC dataset in our study had a mean coverage of 3X compared to 5X of the aforementioned studies. Nevertheless, both datasets in our study were able to successfully separate full siblings from more distantly related animals as shown from their respective distributions of genomic relationships.

### 4.3. Association Analysis

Our association study for length and log_2_ transformed condition factor (K) confirmed that the density of the marker set is crucial when conducting genome-wide regression studies in aquatic animal populations [49,50,51]. All SNPs of the high coverage set failed to reach the genome-wide adjusted significance threshold for both studied traits. SNPs from the low coverage dataset failed to significantly associate with body length but eight of them were identified as significantly associated with log_2_K. Further analysis showed that some of those loci are located in genomic regions containing genes that are affiliated with development and metabolism. Among them, a SNP was located 570 bp upstream of the *apolipoprotein M* (apom) gene, which has been previously associated with obesity in mice [52,53] and humans [54,55]. Moreover, two of the significant SNPs were located in or near genes that code for transcription factors such as klf7 and nkx2.7. Genetic studies on QTLs of condition factor in Arctic charr have been previously conducted [56,57,58], but to our knowledge the current article is the first to present genetic associations using genomic approaches. The authors acknowledge though, that the association study was performed on small sample sizes, and further validation regarding the putative role of the above SNPs would be required. Nevertheless, it should be noted that the studied sample consisted of full-sibling families from a closed breeding nucleus, meaning that the probability of detecting QTLs is higher compared to scenarios where distant related individuals are used.

## 5. Conclusions

Both deep and shallow coverage genotyping by sequencing strategies provides valuable genomic information that can be incorporated in fish breeding programs. While deep coverage results in high quality but less dense genotypes, shallow coverage provides higher genotypic density with a greater proportion of missing data and higher uncertainty. The first strategy performed better in genetic diversity and population structure analyses, while low coverage genotyping proved to be more informative regarding genome-wide regression for association, with phenotypic traits demonstrating the potential of both approaches in aquaculture breeding schemes.

## Figures and Tables

**Table 1:** SNPs significantly associated with the logarithmically transformed condition factor and nearby candidate genes. * SNP located within 1 kb of gene, ** SNP located in gene.

Linkage Group	SNP Position (bp)	Unadjusted *p*-Value	BH Adjusted *p*-Value	Annotated or Predicted Genes within 20 kb on Either Side
NC_036876.1	6,904,331	2.317911 × 10^−9^	8.859984 × 10^−5^	LOC111960292 **
NC_036872.1	16,812,304	1.710431 × 10^−8^	3.268976 × 10^−4^	nkx2.7, LOC111957894, LOC111957491
NC_036874.1	10,834,663	1.568166 × 10^−7^	1.998052 × 10^−3^	apom *, LOC111959084, LOC111958785
NW_019945418.1	7585	2.161829 × 10^−6^	2.030243 × 10^−2^	-
NC_036874.1	13,345,514	2.655718 × 10^−6^	2.030243 × 10^−2^	-
NC_036869.1	2,707,853	4.399173 × 10^−6^	2.761334 × 10^−2^	LOC111955246 *, pex1
NC_036839.1	21,199,964	5.056859 × 10^−6^	2.761334 × 10^−2^	klf7 **
NC_036839.1	17,537,842	8.506600 × 10^−6^	4.064454 × 10^−2^	LOC111972823 **

## Data Availability

The sequence reads in the form of fastq files have been deposited to the National Centre for Biotechnology Information (NCBI) and are publicly available under project ID PRJNA705295.

## Supplementary Materials

The following are available online at https://www.mdpi.com/2076-2615/11/3/899/s1: File S1 containing the barcode combinations that were used for the SbfI-SphI dataset. File S2 containing the barcode combinations that were used for the PstI-NlaIII dataset. Figure S1 depicting the family predictions using DAPC for varying levels of MAF.
