# Supplementary figures and images for: Genotyping Strategies Using ddRAD Sequencing in Farmed Arctic Charr (Salvelinus alpinus)
Source: Animals (Basel). 2021 Mar 21;11(3):899. doi: 10.3390/ani11030899 (PMC8004150; doi:10.3390/ani11030899)

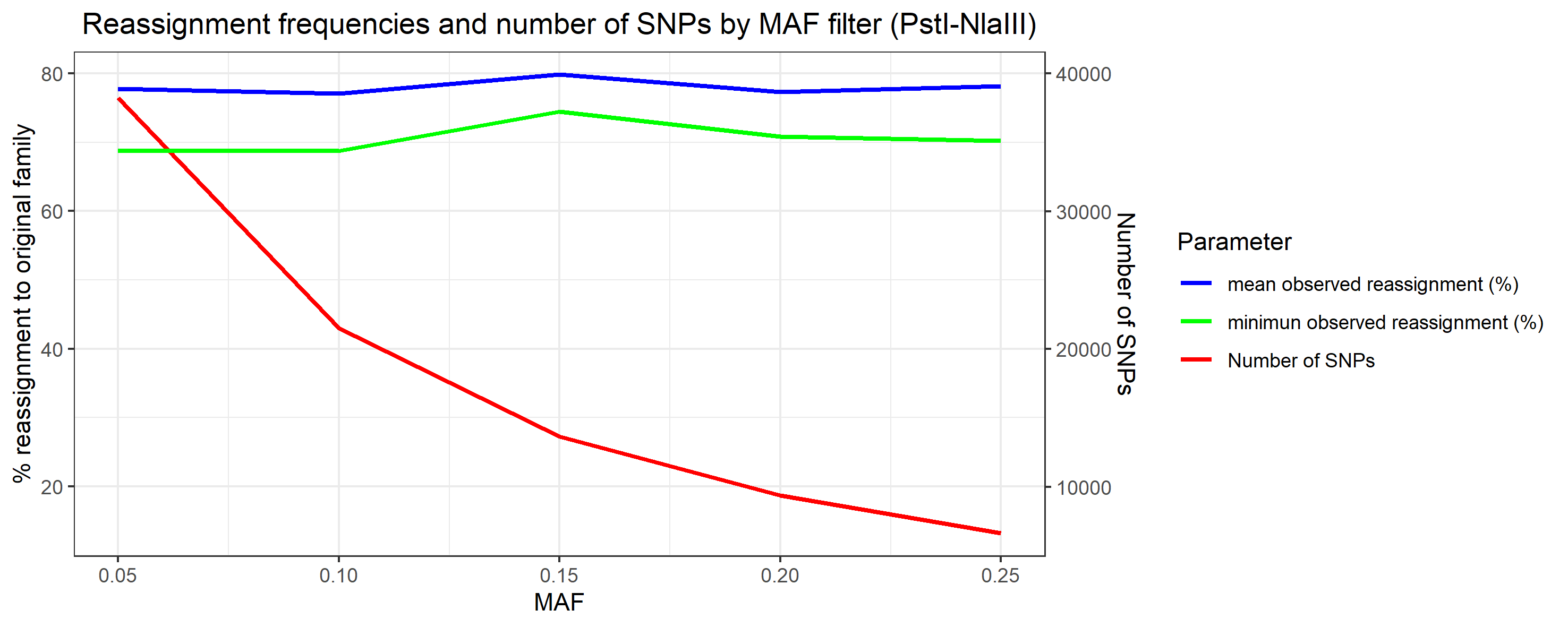

Supplement: Supplementary file 1 [file animals-11-00899-s001.zip › Figure S1.tiff]
